# Renal Safety Profile of EGFR Targeted Therapies: A Study from VigiBase^®^ the WHO Global Database of Individual Case Safety Reports

**DOI:** 10.3390/cancers13235907

**Published:** 2021-11-24

**Authors:** Alexandre Crosnier, Chadi Abbara, Morgane Cellier, Laurence Lagarce, Marina Babin, Delphine Bourneau-Martin, Marie Briet

**Affiliations:** 1 Department of Pharmacology-Toxicology and Pharmacovigilance, University Hospital of Angers, 4 Rue Larrey, 49100 Angers, France; alexandre.crosnier@etud.univ-angers.fr (A.C.); chadi.abbara@chu-angers.fr (C.A.); morgane.cellier@chu-angers.fr (M.C.); lalagarce@chu-angers.fr (L.L.); marina.babin@chu-angers.fr (M.B.); debourneau@chu-angers.fr (D.B.-M.); 2 Département de Médecine, Faculté de Médecine d’Angers, Université d’Angers, 49035 Angers, France; 3 Research Institute MitoVasc, UMR CNRS 6214 INSERM 1083, University of Angers, 49100 Angers, France

**Keywords:** anti-EGFR drugs, adverse drug effect, renal toxicity, pharmacovigilance

## Abstract

**Simple Summary:**

Drugs targeting the epithelial growth factor receptor (EGFR) are used in pulmonary and digestive cancers and represent major medical progress. In addition to its localization in cancer cells, EGFR can also be found in the kidney. This observation raises the question of the renal toxicity of these drugs. This issue has been addressed in the present study conducted on safety data from the largest international pharmacovigilance database, VigiBase^®^. This study showed that the renal toxicity of these drugs is mainly represented by renal failure in the context of digestive toxicity. A new adverse effect called haemolytic and uremic syndrome or thrombotic microangiopathy has been found for erlotinib, which is the first anti-EGFR drug to obtain market authorisation. This signal has to be confirmed. No other renal toxicity has been found related to anti-EGFR drugs, in particular, neither glomerular nor tubular toxicity.

**Abstract:**

Kidney EGFR expression together with reported cases of glomerular diseases in the context of anti-EGFR drug administration raise concerns about the renal safety profile of these drugs. This issue is addressed in a case/non-case study carried out on VigiBase^®^, the WHO global database of individual case safety reports (ICRS). Disproportionality analysis of renal adverse effects related to the selected anti-EGFR drugs, erlotinib, gefitinib, afatinib, osimertinib, cetuximab and panitumumab, was assessed using the reporting odds ratio (ROR). Nine hundred and eighty-nine ICRSs were included. A signal of disproportionate reporting (SDR) was found for afatinib (ROR = 2.70; 95% CI [2.22–3.29]) and erlotinib (ROR = 1.73; 95% CI [1.46–2.04]) with acute kidney injury, and for afatinib (ROR = 2.41; 95% CI [1.78–3.27]), cetuximab (ROR = 1.42; 95% CI [1.14–1.78]) and erlotinib (ROR = 2.23; 95% CI [1.80–2.77]) with renal failure. The preferred term “diarrhoea” was frequently reported in the included cases. An SDR was found for erlotinib with haemolytic and uremic syndrome (ROR = 4.01; 95% CI [1.80–8.94]) and thrombotic microangiopathy (ROR = 4.94; 95% CI [2.80–8.72]). No SDR was seen for glomerular or tubule-interstitial diseases. This study showed that the anti-EGFR drug renal toxicity is mainly related to renal failure in the context of digestive toxicity.

## 1. Introduction

Anti-epidermal growth factor receptor (EGFR) drugs were one of the first targeted therapies developed in the field of oncology [1], and include monoclonal antibodies and tyrosine kinase inhibitors (TKIs). The monoclonal antibodies, cetuximab and panitumumab, are approved by European Medicines Agency (EMA) and Food and Drug Administration (FDA) for the treatment of wild-type metastatic colorectal cancer, advanced non-small cell lung cancer (NSCLC) and head and neck cancer [2,3]. The main indication for anti-EGFR TKIs is NSCLC [4]. Erlotinib and gefitinib were the first generation of anti-EGFR drugs to be developed. The emergence of resistance to treatment despite a good initial response led to the development of new generations of anti-EGFR TKIs, such as afatinib, a second generation anti-EGFR TKI, and osimertinib which targets the EGFR mutation T790M [5,6].

EGFR is a transmembrane cell receptor with tyrosine kinase activity. The binding of a ligand to the extracellular domain of EGFR induces its activation by homodimerisation or heterodimerisation with one of the other receptors of the ErbB family [7]. Phosphorylated tyrosine residues lead to the activation of several cell-signalling pathways, such as RAS/MAPK [8], PI3K/AKT/mTOR [9,10] and JAK/STAT [11] involved in cell survival, proliferation, metastasis and angiogenesis. The development of anti-EGFR drugs considerably improved the prognosis of the patients. The main adverse effects reported are digestive, hepatic and cutaneous toxicity [12,13,14,15,16,17,18,19].

Several clinical studies showed an EGFR expression in the kidney [20,21] in tubular cells, and to a lesser extent in the glomerular cells [22,23]. This observation suggests that anti-EGFR drugs may be associated with direct renal toxicity. Renal failure is inconstantly mentioned in the summary of product characteristics of anti-EGFR drugs. In the literature, few cases of glomerular diseases have been reported in patients exposed to cetuximab, panitumumab, gefitinib and erlotinib [24,25,26,27]. Therefore, the main objective of this work was to evaluate the renal safety profile of drugs targeting EGFR with a case/non-case study conducted on a large pharmacovigilance database, VigiBase^®^, the World Health Organisation’s (WHO) global database of individual case safety reports (ICRS).

## 2. Materials and Methods

### 2.1. Data Source

This case/non-case study was conducted using VigiBase^®^, the WHO global database of suspected adverse reactions to medicinal products. VigiBase^®^ is the largest pharmacovigilance database in the world, with more than 20 million reports of suspected adverse effects of drugs, and is developed and maintained by the Uppsala Monitoring Centre (UMC), an independent centre for drug safety and scientific research [28]. More than 130 countries have joined this programme since 1968 [29]. Adverse drug reaction (ADR) cases are reported by healthcare professionals, pharmaceutical companies and patients. Data recorded include, among others, patients’ age, sex and medical history, time to onset, drugs taken by the patient, adverse effects. Adverse effects are recorded in VigiBase^®^ using the Medical Dictionary for Regulatory Activities (MedDRA^®^) classification, which was established by The International Council for Harmonisation of Technical Requirements for Pharmaceuticals for Human Use (ICH) [30]. This medical dictionary includes standardised medical terminology to facilitate the sharing of information for medical products [31]. The terms are ranked from the most general to the most specific, according to the following tree structure: system organ class (SOC), high level group term (HLGT), high level term (HLT), preferred term (PT) and lowest level term (LLT).

### 2.2. Study Design

This was a retrospective study based on the data collected from VigiBase^®^. The studied drugs were: cetuximab, panitumumab, gefitinib, erlotinib, afatinib and osimertinib as suspected or interacting. The analysis was performed for each drug from the date of marketing authorisation to 1 December 2020.

For renal ADR, the following HLTs from MedDRA^®^ classification were selected, glomerulonephritis and nephrotic syndrome, nephritis NEC, nephropathies and tubular disorders NEC, renal disorders NEC, renal failure and impairment, renal hypertension and related conditions, renal vascular and ischaemic conditions. All the PTs included in the selected HLTs were the renal effects of interest in this study.

The exclusion criteria were cases reported by non-health professionals, duplicate cases and cases with age or sex unknown. Duplicate cases were identified by the VigiMatch^®^ tool and eliminated [32].

For all the cases, the following data were collected and studied: reported date, country of occurrence, seriousness, notifier, patient age, patient sex, effect onset date, reported drugs of interest, drugs status (suspected or interacting), drugs start date, drugs indication, renal adverse effects of interest and co-reported adverse effects.

### 2.3. Statistical Analysis

A disproportionality analysis was performed using the reporting odd ratios (ROR). The minimum threshold considered for a renal adverse effect to be studied was 5, according to the EMA guidelines [33]. The value of the ROR was obtained according to the formula ROR = (a/b)/(c/d), which gives ad/bc, and the 95% confidence intervals (CI) [34] were calculated with:

$$95\%\;\mathrm{CI}={\;e}^{\log\left(\mathrm{ROR}\right)\pm 1.96\sqrt{\frac{1}{a}+\frac{1}{b}+\frac{1}{c}+\frac{1}{d}}}$$

In these equations, “a” was the number of one ADR renal PT of interest with one drug of interest suspected or interacting, “b” was the number of all other ADR PTs with one drug of interest suspected or interacting, “c” was the number of one ADR renal PT of interest with drugs other than the drug of interest, “d” was the number of all other ADR PTs with other drugs than the drug of interest [34].

The time to onset (TTO) was calculated in days from the date of initiation of treatment and the date of onset of the effect (TTO = effect onset date–drug start date). The graphical representation of cumulative distribution function of drugs by TTO was performed by Monolix^®^. Forest plots of disproportionality were obtained using R-software (R version 3.4.2, R Foundation for statistical, Vienna, Austria).

## 3. Results

### 3.1. Description of the Studied Cases

The study population consisted of 989 patient cases with a median age of 68 years (Table 1). The reported cases involved mainly men (59%). Almost all of the cases were considered serious. When the evolution was reported (*n* = 632), it was unfavourable in one third of the cases with death and persistent renal injury.

Among the studied drugs, erlotinib, afatinib and cetuximab were the most frequently reported suspected drugs, in 30%, 20% and 29% of the cases, respectively, whereas gefitinib and osimertinib were drugs reported in less than 10% of the cases (Table 1). The reporting years for each of the studied drugs are presented in Table 2. The main reported indications were NSCLC and colorectal cancer. Acute kidney injury, renal failure and renal impairment were the three most frequently reported ADR renal PTs, representing respectively 42%, 23% and 11% of the renal effects of interest (Table 1). These effects were found among the top three ADRs for each of the drugs studied (Table 2). Fifteen thrombotic microangiopathy cases have been reported, among them, 12 cases were related to erlotinib (Table 2). Nephrotic syndrome was reported in 14 cases (Table 1).

### 3.2. Disproportionality Analysis and Time to Onset (TTO)

Disproportionality analyses were performed for adverse effects reported in more than five cases for one drug.

**Figure 1:**
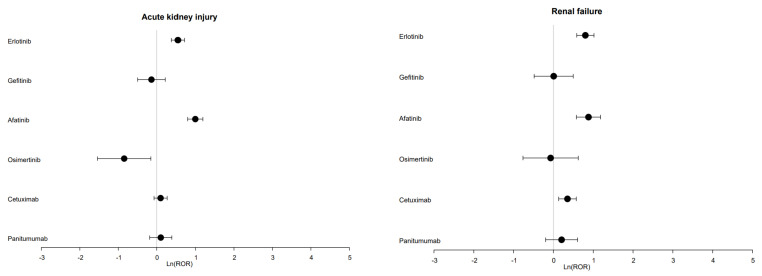
Forest plot of disproportionality (reporting odd ratio) of drugs targeting EGFR and acute kidney injury or renal failure.

**Figure 2:**
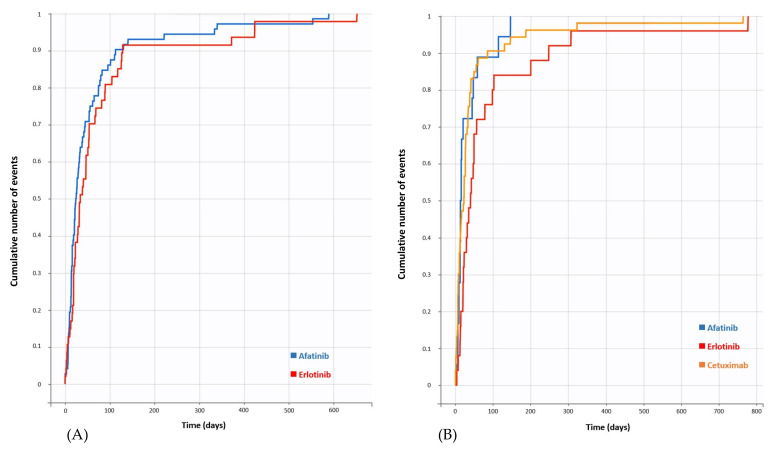
Cumulative distribution function of drugs studied by time to onset of acute kidney injury (**A**) or renal failure (**B**).

With regard to acute kidney injury, a significantly increased ROR was found for afatinib (ROR = 2.70; 95% CI [2.22–3.29]) and erlotinib (ROR = 1.73; 95% CI [1.46–2.04]) (Table S1, Figure 1). The median TTO was 24.5 days (Q1 = 14; Q3 = 57.5) for afatinib with available data for 72 cases, and 34 days (Q1 = 19; Q3 = 75.5) for erlotinib with available data for 47 cases (Figure 2).

In terms of renal failure, a significant disproportionality signal was found for afatinib (ROR = 2.41; 95% CI [1.78–3.27]), cetuximab (ROR = 1.42; 95% CI [1.14–1.78]) and erlotinib (ROR = 2.23; 95% CI [1.80–2.77]) (Table S1, Figure 1). The median TTO was 15 days (Q1 = 11; Q3 = 39) for afatinib with available data for 18 cases, 23 days (Q1 = 8; Q3 = 35) for cetuximab with available data for 53 cases and 41 days (Q1 = 21; Q3 = 79) for erlotinib with available data for 25 cases (Figure 2).

Diarrhoea was frequently noted in association with acute kidney injury PT for afatinib and erlotinib and with renal failure for afatinib, cetuximab and erlotinib (Table 3). 

A disproportionality signal was seen for afatinib, osimertinib and renal impairment (ROR = 1.71; 95% CI [1.16–2.52], ROR = 1.74; 95% CI [1.01–3.01], respectively) (Table S1).

Erlotinib was related with a signal of disproportionate reporting for haemolytic uraemic syndrome (ROR = 4.01; 95% CI [1.80–8.94]) and thrombotic microangiopathy (ROR = 4.94; 95% CI [2.80–8.72]) (Table S1).

Gefitinib and panitumumab were the only drugs with no significant ROR for the renal effects of interest (Table S1).

## 4. Discussion

With the exception of gefitinib and panitumumab, a significant disproportionality signal was found for all the studied drugs targeting EGFR, with at least one of the following: acute kidney injury, renal failure or renal impairment. No disproportionality signal was seen with MedDRA^®^ terms related to specific kidney injury, such as glomerulonephritis, nephrotic syndrome or interstitial diseases. A new safety signal emerged for erlotinib related to haemolytic uraemic syndrome and thrombotic microangiopathy.

Regarding acute kidney injury or renal failure signal, the ADR PT most commonly reported in these cases was diarrhoea. This observation, together with the absence of a significant disproportionality signal for renal diseases, such as glomerulopathy or tubule-interstitial diseases suggest that the most common mechanism of renal failure or acute kidney injury related to anti-EGFR therapies is functional, secondary to dehydration which can be due to a digestive toxicity of these drugs. When available, the time to onset is compatible with this hypothesis. Regarding the mechanism involved, EGFR is expressed in the gastrointestinal tract, mainly found on the basolateral membranes of intestinal epithelial cells [35]. EGFR is involved in the regulation of maintaining mucosal integrity and in the regulation of ionic transport by negative control of intestinal epithelial chloride secretion. The downregulation of chloride secretion is responsible for the passive movement of water through the gastrointestinal lumen. One of the hypotheses put forward for anti-EGFR TKIs is that these drugs would be responsible for blocking the negative regulation of chloride secretion, which could explain the occurrence of diarrhoea [36,37,38]. However, the mechanism responsible for diarrhoea is not fully elucidated and other hypotheses have also been developed, such as direct mucosal damage [36]. Among the three generations of anti-EGFR TKIs, the second generation is associated with the highest incidence of diarrhoea [37]. 

Notably, a disproportionality signal has been shown for erlotinib and haemolytic uraemic syndrome/thrombotic microangiopathy. This signal was not seen with the other anti-EGFR studied drugs. The occurrence of renal thrombotic microangiopathy is well described in the context of VEGF receptor inhibition. Erlotinib is the only anti-EGFR TKI which has a vascular endothelial growth factor receptor-2 (VEGFR2) selectivity, even if the selectivity is low [39]. In addition to a direct inhibitory effect of erlotinib on the VEGF receptor, indirect mechanisms could also be involved, such as a decrease in VEGF expression in the kidney. This hypothesis is supported by the expression of EGFR in peritubular vessel and glomeruli [22,23], and by the inhibitory effect of EGFR blockade on the PI3K/AKT/mTOR signalling pathway that could lead to a decrease in VEGF expression. Indeed, the mTOR protein has a proangiogenic role since it regulates the translation of the hypoxia inducible factor (HIF) which is involved in the stimulation of the expression of VEGF [40]. Another hypothesis could be that erlotinib decreases VEGF expression through the inhibition of the JAK/STAT signalling pathway [41]. Therefore, erlotinib may also have an anti-VEGF effect, directly or indirectly, which could be responsible for thrombotic microangiopathy [26,42,43]. This effect is not reported in the SmPC of the drug and needs to be confirmed by further studies.

Despite experimental studies showing the presence of EGFR in epithelial cells of the distal tubule, collecting tubule and glomeruli [23,24,25,44,45], only few cases of glomerular or tubular and interstitial diseases have been reported in VigiBase^®^, and did not reach the threshold of five cases for disproportionality analyses. Since some anti-EGFR drugs recently obtained market authorisation, further pharmaco-epidemiological studies are needed to evaluate these adverse effects with a longer follow-up.

Limitations were present in this study. Disproportionality analysis does not make it possible to determine a level of risk, but it is limited to the emission of signals. In addition, the information comes from a variety of sources, and the probability that the suspected adverse effect is drug-related is not the same in all cases. The under-reporting of an adverse drug reaction, classically described in pharmacovigilance, is a bias that could affect the results of disproportionality studies. The under-reporting of adverse drug effects varies according to the type of drug and the type of ADR [46]. In addition, the data of VigiBase^®^ were sometimes incomplete, which could constitute an information bias. For example, the TTO could not be calculated for all the cases because the date of treatment initiation or the precise date of adverse drug effects were missing. Eventually, it is possible that the patients exposed to the drug of interest may be more (or less) at risk of effects of interest than those exposed to other drugs [34].

## 5. Conclusions

This case/non-case study carried out on VigiBase^®^, the WHO global database of individual case safety reports (ICRS), confirmed that renal failure is an adverse effect of afatinib, erlotinib and osimertinib, mostly in the context of diarrhoea. A signal of renal failure not mentioned in the EMA SmPC for cetuximab was identified. No glomerular disease signal was identified. This ADR could be reassessed with a longer follow-up, since some anti-EGFR drugs recently obtained marketed authorisation. Finally, a signal of haemolytic uraemic syndrome/thrombotic microangiopathy emerged with erlotinib and needs to be confirmed.

## Figures and Tables

**Table 1:** Main characteristics of the 989 studied cases. PT: preferred term.

Parameters	*n*	%
Patient’s	989	
Age, years, median (Q1–Q3)	68 (60–74)	
Sex, Female/Male	402/587	40.6/59.4
Seriousness		
Serious	950	96.1
Not serious	31	3.1
Unknown	8	0.8
Outcome		
Death	82	8.3
Not recovered/not resolved	116	11.7
Recovered/resolved with sequelae	15	1.5
Recovered/resolved	419	42.4
Unknown	357	36.1
Top 5 reporting countries		
United States of America	326	33.0
Japan	181	18.3
Germany	139	14.1
France	81	8.2
Belgium	35	3.5
Tyrosine kinase inhibitors and monoclonal antibodies of interest	999	
Erlotinib	303	30.3
Gefitinib	75	7.5
Afatinib	199	19.9
Osimertinib	31	3.1
Cetuximab	290	29.0
Panitumumab	101	10.1
Top 10 reporting MedDRA PT events of interest	1079	
Acute kidney injury	458	42.4
Renal failure	252	23.4
Renal impairment	117	10.8
Renal disorder	41	3.8
Chronic kidney disease	20	1.9
Prerenal failure	15	1.4
Fluid retention	15	1.4
Thrombotic microangiopathy	15	1.4
Nephrotic syndrome	14	1.3
Renal tubular necrosis	12	1.1

**Table 2:** Main indications and adverse drug effects reported for the 6 studied drugs targeting EGFR. PT: preferred term.

Characteristics of the Drugs	Erlotinib *n* = 303	Gefitinib *n* = 75	Afatinib *n* = 199	Osimertinib *n* = 31	Cetuximab *n* = 290	Panitumumab *n* = 101
**Reporting year, *n* (%)**						
Before 2010	61 (20.1)	24 (32.0)	0 (0.0)	0 (0.0)	86 (29.7)	4 (4.0)
2010	32 (10.6)	2 (2.7)	3 (1.5)	0 (0.0)	20 (6.9)	8 (7.9)
2011	35 (11.6)	7 (9.3)	3 (1.5)	0 (0.0)	22 (7.6)	9 (8.9)
2012	23 (7.6)	2 (2.7)	8 (4.0)	0 (0.0)	7 (2.4)	1 (1.0)
2013	27 (8.9)	5 (6.7)	18 (9.0)	0 (0.0)	12 (4.1)	8 (7.9)
2014	41 (13.5)	10 (13.3)	11 (5.5)	0 (0.0)	41 (14.1)	8 (7.9)
2015	17 (5.6)	4 (5.3)	46 (23.1)	0 (0.0)	25 (8.6)	9 (8.9)
2016	20 (6.6)	6 (8.0)	37 (18.6)	1 (3.2)	13 (4.5)	16 (15.8)
2017	23 (7.6)	5 (6.7)	18 (9.0)	6 (19.4)	23 (7.9)	13 (12.9)
2018	12 (4.0)	3 (4.0)	31 (15.6)	15 (48.4)	15 (5.2)	14 (13.9)
2019	6 (2.0)	5 (6.7)	13 (6.5)	6 (19.4)	20 (6.9)	5 (5.0)
2020	6 (2.0)	2 (2.7)	11 (5.5)	3 (9.7)	6 (2.1)	6 (5.9)
Top 3 reported indications (MedDRA PT), *n* (%)	Non-small cell lung cancer 78 (25.7) Lung neoplasm malignant 20 (6.6) Lung adenocarcinoma 17 (5.6)	Lung adenocarcinoma 12 (16.0) Non-small cell lung cancer 11 (14.7) Lung neoplasm malignant 4 (5.3)	Non-small cell lung cancer 55 (27.6) Lung adenocarcinoma 35 (17.6) Malignant neoplasm of bronchus and lung 18 (9.0)	Non-small cell lung cancer 18 (58.1) Lung cancer 3 (9.7) Non-small cell lung cancer metastatic 2 (6.5)	Non-small cell lung cancer 23 (7.9) Metastatic colorectal cancer 22 (7.6) Colorectal cancer 17 (5.9)	Colon cancer 17 (16.8) Metastatic colorectal cancer 15 (14.9) Colorectal cancer 10 (9.9)
Unknown indication, *n* (%)	32 (10.6)	14 (18.7)	20 (10.1)	3 (9.7)	27 (9.3)	5 (5.0)
Top 5 reported adverse drug reactions (MedDRA PT), *n* (%)	Acute kidney injury 139 (45.9) Renal failure 83 (27.4) Renal impairment 26 (8.6) Renal disorder 15 (5.0) Thrombotic microangiopathy 12 (4.0)	Acute kidney injury 30 (40.0) Renal failure 16 (21.3) Renal impairment 9 (12.0) Renal disorder 3 (4.0) Nephrotic syndrome 3 (4.0)	Acute kidney injury 101 (50.8) Renal failure 42 (21.1) Renal impairment 26 (13.1) Renal disorder 11 (5.5) Prerenal failure 10 (5.0)	Renal impairment 13 (41.9) Renal failure 8 (25.8) Acute kidney injury 8 (25.8) Renal disorder 2 (6.5) Nephrotic syndrome 1 (3.2)	Acute kidney injury 133 (45.9) Renal failure 79 (27.2) Renal impairment 32 (11.0) Renal disorder 8 (2.8) Renal tubular disorder 4 (1.4)	Acute kidney injury 47 (46.5) Renal failure 24 (23.8) Renal impairment 11 (10.9) Nephrotic syndrome 4 (4.0) Nephropathy 3 (3.0)

**Table 3:** Associated adverse drug reactions reported in acute kidney injury and renal failure cases. ADR: adverse drug reaction; AKI: acute kidney injury; RF: renal failure.

Variation	Acute Kidney Injury		Renal Failure	
	Number of AKI Cases	Top 5 Associated ADRs, *n* (% of AKI Cases)	Number of RF Cases	Top 5 Associated ADRs, *n* (% of RF Cases)
Erlotinib	139	Diarrhoea, 50 (36.0) Dehydration, 33 (23.7) Vomiting, 23 (16.5) Nausea, 19 (13.7) Anaemia, 16 (11.5)	83	Diarrhoea, 25 (30.1) Dehydration, 13 (15.7) Rash, 9 (10.8) Vomiting, 9 (10.8) Dyspnoea, 9 (10.8)
Afatinib	101	Diarrhoea, 56 (55.4) Dehydration, 20 (19.8) Vomiting, 19 (18.8) Decreased appetite, 13 (12.9) Nausea, 10 (9.9)	42	Diarrhoea, 35 (83.3) Dehydration, 19 (45.2) Vomiting, 8 (19.0) Rash, 7 (16.7) Nausea, 5 (11.9)
Cetuximab	-		79	Diarrhoea, 20 (25.3) Dehydration, 13 (16.5) Sepsis, 9 (11.4) Fatigue, 8 (10.1) Blood creatinine increased, 7 (8.9)

## Data Availability

The data presented in this study are available on request from the corresponding author. The request should be accompanied by a research protocol. The data are not publicly available due to European ethical and legal restrictions.

## Supplementary Materials

The following are available online at https://www.mdpi.com/article/10.3390/cancers13235907/s1, Table S1: ROR from standard disproportionality analysis with threshold (*n* ≥ 5).
