# Supplementary material for: Renal Safety Profile of EGFR Targeted Therapies: A Study from VigiBase® the WHO Global Database of Individual Case Safety Reports
Source: Cancers (Basel). 2021 Nov 24;13(23):5907. doi: 10.3390/cancers13235907 (PMC8657199; doi:10.3390/cancers13235907)
Supplement: Supplementary file 1 [file cancers-13-05907-s001.zip › cancers-1409705-supplementary.pdf]

Table S1. Reporting odds ratio from standard disproportionality analyses. PT: preferred term

DRUGS / EFFECTS (MedDRA PT)	Erlotinib			Gefitinib			Afatinib			Osimertinib			Cetuximab			Panitumumab		
	n	ROR	95% CI	n	ROR	95% CI	n	ROR	95% CI	n	ROR	95% CI	n	ROR	95% CI	n	ROR	95% CI
Acute kidney injury	139	**1,73**	1,46 - 2,04	30	0,87	0,61 - 1,25	101	**2,70**	2,22 - 3,29	8	**0,43**	0,21 - 0,86	133	1,11	0,93 - 1,31	47	1,11	0,83 - 1,48
Prerenal failure							10	**45,24**	24,14 - 84,80									
Chronic kidney disease	9	1,19	0,62 - 2,28				7	1,97	0,94 - 4,15									
Haemolytic uraemic syndrome	6	**4,01**	1,80 - 8,94															
Thrombotic microangiopathy	12	**4,94**	2,80 - 8,72															
Renal tubular necrosis	6	1,56	0,70 - 3,48															
Fluid retention	9	0,72	0,38 - 1,39															
Hepatorenal syndrome	5	**5,80**	2,41 - 13,97															
Nephropathy toxic	5	1,04	0,43 - 2,50															
Renal disorder	15	**1,89**	1,14 - 3,13				11	**2,96**	1,64 - 5,35				8	0,68	0,34 - 1,35			
Renal failure	83	**2,23**	1,80 - 2,77	16	1,01	0,62 - 1,65	42	**2,41**	1,78 - 3,27	8	0,93	0,47 - 1,87	79	**1,42**	1,14 - 1,78	24	1,23	0,82 - 1,83
Renal impairment	26	0,80	0,54 - 1,17	9	0,65	0,34 - 1,25	26	**1,71**	1,16 - 2,52	13	**1,74**	1,01 - 3,01	32	**0,66**	0,47 - 0,93	11	0,65	0,36 - 1,17
